# Parasitic Infections in Hematopoietic Stem Cell Transplantation

**DOI:** 10.4084/MJHID.2016.035

**Published:** 2016-07-01

**Authors:** Isidro Jarque, Miguel Salavert, Javier Pemán

**Affiliations:** 1 Hematology Department. Hospital Universitario y Politécnico La Fe, Valencia, Spain; 2 Infectious Diseases Unit. Hospital Universitario y Politécnico La Fe, Valencia, Spain; 3 Microbiology Department. Hospital Universitario y Politécnico La Fe, Valencia, Spain

## Abstract

Parasitic infections are rarely documented in hematopoietic stem cell transplant recipients. However they may be responsible for fatal complications that are only diagnosed at autopsy. Increased awareness of the possibility of parasitic diseases both in autologous and allogeneic stem cell transplant patients is relevant not only for implementing preventive measures but also for performing an early diagnosis and starting appropriate therapy for these unrecognized but fatal infectious complications in hematopoietic transplant recipients. In this review, we will focus on parasitic diseases occurring in this population especially those with major clinical relevance including toxoplasmosis, American trypanosomiasis, leishmaniasis, malaria, and strongyloidiasis, among others, highlighting the diagnosis and management in hematopoietic transplant recipients.

## Introduction

Parasitic diseases continue to have a significant impact on health, quality of life, and economic development wherever they are endemic. In addition, medical advances such as solid organ transplantation compromise the immune system of patients, which render them vulnerable to previously rare opportunistic organisms or to the exacerbation of latent parasitic infections.^1^,^2^

Parasitic diseases are the most understudied of all infections related to hematopoietic stem cell transplantation (HSCT).^3^–^5^ Parasitic diseases are frequently neglected in this setting in spite of causing life-threatening complications. As a consequence management recommendations in this field are mainly based on expert opinions provided that prospective trials or randomized studies are lacking.

In this review, we will focus on parasitic diseases occurring in HSCT recipients especially those with major clinical relevance (Table 1). A better knowledge of these entities would help to improve prognosis in these potentially fatal complications in which a high level of suspicion could be critical for a proper management.

### Toxoplasmosis

Toxoplasmosis is a zoonotic illness due to infection with the protozoan *Toxoplasma gondii*. Although toxoplasmosis occurs worldwide it is more common in patients from endemic regions, including France and the moist tropical areas of Latin America and sub-Saharan Africa, where the prevalence may approach 90%. Risk factors for primary infection include ingestion of cysts in undercooked meat (including beef and lamb) or contaminated soil, contact with oocysts in feline feces, maternal-fetal transmission, or via blood transfusion or transplant from an infected donor.^6^ Infection in HSCT recipients can occur through ingestion of contaminated food or water, after receiving an infected allograft, or by reactivation of latent infection.^7^–^15^

Transplant patients with toxoplasmosis can present with fever, lymphadenopathy, hepatosplenomegaly, meningitis, brain abscess, chorioretinitis, pneumonitis, myocarditis, hepatitis, pancytopenia or disseminated disease. Symptoms often present within 3 months post-transplant but later presentations can be seen, particularly after discontinuation of chemoprophylaxis, or even interruption of the anti-*Pneumocystis* prophylaxis with cotrimoxazole, which may exert a protective role.

Diagnosis is usually based on the clinical syndrome plus a positive antibody. Definite diagnosis requires the identification of tachyzoites in tissue, or positivity of nucleic acid amplification tests (NAAT) in cerebrospinal fluid (CSF) and/or infected tissues.^16^ Multiple ring-enhancing lesions in the basal ganglia or cerebrum on neuroimaging, especially with anti-Toxoplasma IgG seropositivity, is suggestive of central nervous system (CNS) toxoplasmosis and is enough to start treatment. HSCT recipients often show a variable enhancement pattern, with the lesion enhancement inversely correlated with the severity of immunosuppression.^17^ Brain biopsy should be considered in non-responding patients, as the radiographic differences with other infections or malignancies are not sufficiently accurate. CSF may have a mild mononuclear pleocytosis and/or an elevated protein. Identification of Toxoplasma DNA by NAAT in CSF has a high specificity (96–100%) but the sensitivity is more limited (52–98%). Rarely tachyzoites can be seen on centrifuged CSF samples after Giemsa staining.

Myocarditis may present with heart failure and diagnosis is made by seeing tachyzoites on myocardial biopsy. Chorioretinitis often presents with scotoma, blurred vision, pain or photophobia. Raised, yellow-white, cottony lesions in a nonvascular distribution (unlike the perivascular exudates of CMV retinitis) are seen and vitreal inflammation may be present on fundoscopic examination. The pulmonary disease often presents with fever, dyspnea, cough, and reticulonodular infiltrates on chest imaging. This pattern of disease may be indistinguishable from *Pneumocystis jirovecii* pneumonia but *T. gondii* tachyzoites are identified in the bronchoalveolar lavage (BAL) fluid. Serum levels of lactate dehidrogenase (LDH), unlike *P. jirovecii* pneumonia, may be extremely high (above 1000 U/L). Although rare, cutaneous toxoplasmosis has been reported after HSCT.^18^

Treatment for active toxoplasmosis includes induction therapy with pyrimethamine (200 mg po ×1 dose, then 75 mg/day po) (plus folinic acid) and sulfadiazine (1 g if <60 kg; 1.5 g if >=60 kg po, q6h) plus folinic acid (10–25 mg/day po). Induction therapy is usually given for at least six weeks depending on the response to treatment. In case of allergy or intolerance to sulfadiazine, it can be used clindamycin (600–900 mg q6h) (Table 2).

In case of worsening of clinical symptoms or brain imaging following an initial improvement after 14–21 days of treatment, it should be considered a possible diagnosis of the immune-reconstitution inflammatory syndrome (IRIS), and practice of a puncture or biopsy of the lesion to rule out other etiologies aside from the CNS toxoplasmosis.

Induction therapy should be followed by chronic suppressive therapy (secondary prophylaxis) to eradicate the encysted form of the parasite and prevent recrudescence of disease. Chronic suppressive therapy with reduced toxicity medication, such as cotrimoxazole may be considered.^19^

The routine use of cotrimoxazole for post-HSCT prophylaxis has decreased the risk of toxoplasmosis and is currently the routine prophylaxis against this parasite. Multiple studies support the efficacy of primary prophylaxis with cotrimoxazole, although the optimal dose and duration of cotrimoxazole remain unclear. Many studies showed successful prophylaxis using cotrimoxazole (160 mg of trimethoprim, 800 mg of sulfamethoxazole) thrice weekly for varying durations (range 3 months to lifelong). In patients with HIV/AIDS, cotrimoxazole one tablet daily is recommended as first line prophylaxis. An alternative to cotrimoxazole is dapsone plus pyrimethamine (plus folinic acid). Atovaquone with or without pyrimethamine (plus folinic acid) has not been well studied but is also considered a likely effective alternative regimen.

Other possible alternatives in immunocompromised hosts included combination of pyrimethamine (and folinic acid) with atovaquone, clarithromycin, azithromycin or dapsone. Folinic acid must be added to all regimens containing pyrimethamine, can be raised the dose to 50 mg/day in case of myelotoxicity by pyrimethamine.

To avoid primary infection, transplant recipients should avoid contact with undercooked meat, soil, water or animal feces that might contain *T. gondii* cysts.

## Chagas disease (American trypanosomiasis)

Chagas disease is caused by the protozoan *Trypanosoma cruzi* and infection is transmitted to humans in Latin America by inoculation of infected feces from triatomine bugs.^20^ However, *T. cruzi* infection can also be transmitted via blood transfusion, infected mother to fetus, oral ingestion, or transplantation. Chagas disease is endemic in most Latin-American countries where 8–9 million people are currently living with infection and 2–5 million people have chagasic cardiomyopathy.

In the last decades, increasing international migration and travel from Latin America to Europe have favored the emergence of Chagas disease outside its traditional boundaries. In the absence of the vector, one of the potential modes of transmission of Chagas disease in non-endemic regions is through blood and blood products. As a consequence cases of Chagas disease have been reported in HSCT recipients.^21^–^24^

Human disease has two distinct phases: the acute phase and the chronic infection. In the normal host, the acute illness ( a febrile syndrome with lymphadenopathy and hepatosplenomegaly) usually resolves spontaneously even if untreated. However, without specific treatment, the infection persists in spite of strong evidence of immunity and patients become chronically infected with the parasite. The phase of clinical latency can last 10–30 years or lifelong and approximately 30% of patients will develop an irreversible disease in heart (27%), esophagus and colon (6%) and peripheral nervous system (3%). *T. cruzi* seronegative recipients of seropositive donors might develop an acute infection after transplantation. Clinical manifestations can include fever, malaise, anorexia, hepatosplenomegaly and acute myocarditis with a mean time to symptom onset of 112 days (range 23–240 days).^25^

Diagnosis of chronic infection is made by detection of antibodies to *T. cruzi* antigens, most commonly by enzyme immunoassay (EIA) or immunofluorescence immunoassay (IFA) methods. No single test has sufficient sensitivity or specificity to be relied on alone for clinical diagnosis. Therefore, two serological tests based on different antigens and/or techniques should be used to increase the accuracy of the diagnosis. When discordant testing occurs, a third test should be employed.

In transplant recipients with acute infection and those with chronic *T. cruzi* infection where there is a concern for reactivation, serological testing has limited utility. Direct parasitological test methods for diagnosis include microscopy of the fresh buffy coat preparations, Giemsa-stained peripheral blood smears, and NAAT of whole blood or tissue from a biopsy. PCR techniques provide the most sensitive testing and can often identify positive results days to weeks before circulating trypomastigotes are detectable by microscopy of peripheral blood smear and buffy coat preparations. Patients with chronic *T. cruzi* infection may have positive NAAT in blood in the absence of disease reactivation and may not be helpful.

Monitoring for infection after transplant from a seropositive donor to a seronegative recipient and after transplantation in a recipient with chronic *T. cruzi* infection is recommended so that treatment can be initiated before the development of clinically significant disease. Monitoring can be accomplished by NAAT of blood for *T. cruzi* (when available) and review of peripheral blood for parasitemia weekly for 2 months post-transplant, every two weeks for the third month, then monthly afterwards for a period to be determined by the specific clinical scenario (intensified immunosuppression, unexplained fever, graft versus host disease).

Treatment is recommended for patients with evidence of reactivation of chronic infection or acute infection post-transplantation. Two drugs are available for treatment of Chagas disease, nifurtimox and benznidazole.^26^,^27^ Both drugs have significant toxicity: benznidazole is frequently associated with rash and a dose-dependent peripheral neuropathy while nifurtimox is associated with gastrointestinal (anorexia, weight loss, nausea) and CNS (irritability, insomnia, tremors) symptoms. Benznidazole (5–7 mg/kg/day po bid × 60 days) is better tolerated among transplant recipients and has fewer drug interactions when compared to nifurtimox and therefore, benznidazole is generally preferred for first-line treatment (Table 2). Posaconazole has shown anti-Trypanosoma activity in patients with chronic Chagas disease. However, in a randomized clinical trial to assess the efficacy and safety of posaconazole versus benznidazole administered for 60 days in immunocompetent adults with chronic *T. cruzi* infection, significantly more patients in the posaconazole group had treatment failure during follow-up (higher rate of relapse). The role of this antifungal agent with anti-Trypanosoma activity in immunosuppressed patients is unknown at present.^28^

Prophylactic therapy in asymptomatic patients is not recommended and preemptive monitoring with NAAT and peripheral smear are preferred to determine the need for treatment. Confirmation of infection (or its absence) has significant implications for long-term management.

## Leishmaniasis

Leishmaniasis is caused by a heterogeneous group of protozoan parasites belonging to the genus *Leishmania* and presents with a variety of different clinical syndromes. The infection is acquired primarily through the bite of an infected female sandfly (genus *Phlebotomus* or *Lutzomyia*). Occasionally the disease is transmitted by transfusion, transplantation or, in drug addict patients, by sharing contaminated needles or syringes. It is also possible maternal-fetal vertical transmission. It is estimated that 350 million people are at risk of acquiring the infection and that 12 million may be infected. Leishmaniasis is found in tropical and subtropical climates and is endemic in the Mediterranean Basin countries. The disease may appear as late as 30 years after the initial infection. Therefore, even a distant exposure needs to be considered for differential diagnosis.

Derangement of host cellular immunity is a significant risk factor for the development of symptomatic and severe infections and for increased mortality in patients with leishmaniasis. Leishmaniasis is the paradigm of imbalance between the immune responses of type Th1/Th2 and Th17/Treg, with functional disruption of granuloma. In most immunocompetent hosts, the *Leishmania* spp*.* infection is asymptomatic; however, viable organisms remain latent for the life of the host. The main potential mechanisms of acquisition in transplant recipients are a primary infection or reactivation of latent infection, both after transplantation.

**Figure 1:**
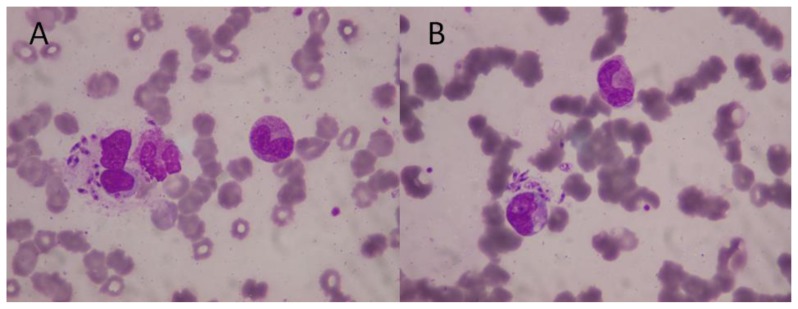
*Leishmania infantum* in bone marrow smears. A: Macrophage containing amastigote forms (Leishman-Donovan bodies). B: Apparently extracellular parasites (May-Grünwald-Giemsa staining 600X)

Clinical manifestations of disease vary based on the infecting organism and host immune response. Visceral leishmaniasis is caused by *L. donovani* complex (*L. donovani*, *L. infantum* and *L. chagasi*) and the clinical features are similar to those of immunocompetent patients. Patients suffer from fever, hepatosplenomegaly and pancytopenia. Median time to onset was 30 days post-transplant (range, 7 days to 5 months) but reactivations as far as 55 and 96 months posttransplantation have been reported.^29^ Information about HSCT patients is limited mainly to case reports.^30^–^35^ Microscopic examination of Giemsa stained bone marrow aspirate/biopsy (Figure 1),^36^ splenic aspirate, culture in special media (NNN and others) and NAAT are the gold standards for diagnosis of visceral leishmaniasis. Occasionally the diagnosis can be made from a biopsy of other tissues such as lymph node, liver or intestine, or by means of fine needle spleen puncture. Serological testing for visceral leishmaniasis is highly sensitive in transplant recipients but serology cannot distinguish between prior exposure and active infection and may cross-react with other protozoa. Urinary antigen detection shows high sensitivity but poor specifity for the diagnosis of visceral leishmaniasis.^37^

Drugs with efficacy in the treatment of visceral leishmaniasis include amphotericin B, pentavalent antimony, paromomycin, and miltefosine but liposomal amphotericin B (3 mg/_Kg_ IV once daily, days 1–5 and days 14, 21) has been shown to be the most efficacious treatment for this disease in immunocompetent patients.^38^ In immunocompromised hosts (Table 2) the FDA approved regimen is liposomal amphotericin B 4 mg/kg daily on days 1–5, 10, 17, 24, 31 and 38 (total of 40 mg/kg).^39^ Cure rates with liposomal amphotericin B in immunocompromised patients approach the same success seen in immune competent hosts. Miltefosine (2,5 mg/kg/day, maximum daily dose of 150 mg) is an oral treatment option in case of amphotericin B intolerance or toxicity. Secondary prophylaxis with intermittent dosing of this drug, every 3 to 4 weeks as in AIDS, may be useful for preventing relapse. Tested combinations (of amphotericin B and miltefosine) may allow shortening the duration of treatment. In refractory cases, with no response or frequent relapses due to major problems of cellular immunity, it has tested the addition of interferon-gamma with varying results, but this option can involve risks in transplant patients.

Data are lacking to determine if screening potential transplant recipients for visceral leishmaniasis would be beneficial. However, those known to be seropositive at the time of transplant should be monitored closely for signs and symptoms of reactivation of infection. Given the limited data on potential donor-derived infection, donor screening cannot be recommended.

## Malaria

Malaria represents an immense health problem in developing countries where it is the cause of more than 300 million acute cases and over 1 million deaths per year. It is transmitted to humans mostly through the bite of the female *Anopheles* mosquito. Blood transfusions and organ transplantation are responsible for some cases in endemic areas and occasionally in countries with large immigrant populations. The disease does not produce protective immunity, but some degree of resistance to clinically severe hyperinfection is achieved through successive exposure and through the persistence of plasmodia in the liver, the microvasculature and the blood stream. This incomplete acquired immunity is unable to eradicate the infection completely but explains the lack of detectable parasitemia and the higher incidence of asymptomatic disease in adults from endemic regions. This poses a problem at the time of blood or organ donation when the epidemiological background is not appropriately investigated.

Many cases of malaria have been described in transplant recipients. In developed countries the disease is seldom seen but it should be considered when caring for a transplanted patient who has resided or visited areas where the disease is endemic (or has received a transplant from a donor who has been in endemic areas) and presents with an unexplained febrile illness. The four different main plasmodia species that infect humans, *Plasmodium ovale*, *P. vivax, P. malariae* and *P. falciparum*, have all been diagnosed in transplant recipients. Fever is the most frequent presenting symptom, but it has not always the typical paroxysmal or cyclic pattern. The main risk in immunosuppressed patients is the development of severe forms of malaria, with multiple organ failure (respiratory distress syndrome, cardiomyopathy, renal and liver failure, etc.) or involvement of CNS.

Malaria is classically diagnosed by microscopic observation of Giemsa stained thick and thin blood smears with the determination of percent parasitemia. Rapid diagnostic tests are available by using dipsticks and allow the detection of specific plasmodia antigens in clinically significant malarial infections. Alternative diagnostic techniques that are recommended in some circumstances to screen blood donors include antigen detection immunochromatographic assay (a rapid diagnostic test); immunofluorescent-assay techniques for species-specific enzymes and NAAT. In most post-transplant cases, the diagnosis was made by the identification of the parasite in blood smears in febrile patients with unexplained hemolysis and thrombocytopenia.

Specific treatment of malaria relies on the use of anti-*Plasmodium* drugs. The identification of plasmodia species, the knowledge of their geographical distribution and their susceptibility patterns are essential. *P. vivax*, *P. malariae*, *P. ovale* and uncomplicated *P. falciparum* infection in chloroquine-susceptible regions should be treated with chloroquine phosphate (1 g −600 mg base- po, them 0.5 g in 6 h, then 0.5 g daily × 2 days; total 2500 mg) or hydroxychloroquine (800 mg salt −620 mg base- po, followed by 400 mg salt po at 6, 24, 48 h; total: 2000 mg salt). However, resistance to chloroquine has been described from Oceania for *P. vivax.* Uncomplicated *P. falciparum* infection acquired in a chloroquine resistant region can be treated with an artemisinin combination therapy (artemether-lumefantrine 4 tablets −80 mg/480 mg- as a single dose, then 4 tablets again after 8 h, then 4 tablets every 12 h for 2 days), atovaquone-proguanil (4 tablets −1000 mg/400 mg- po in a single dose daily × 3 days), quinine-based regimen (quinine sulfate 650 mg pot id × 3 days, plus doxycycline 100 mg po bid), or mefloquine (750 mg po × 1 dose, then 500 mg po × 1 dose 6–12 h later). Severe cases of *P. falciparum* infection should be treated with intravenous artesunate followed by doxycycline, atovaquone-proguanil or mefloquine (Table 2). When artesunate is not available, intravenous quinine or quinidine plus doxycycline, tetracycline or clindamycin should be given. Primaquine phosphate (2 tablets po once daily × 14 days) should be used to prevent relapse of *P. vivax* and *P. ovale* (after rule out G6PD deficiency).

Malaria is potentially fatal in the transplant recipient. Early diagnosis and conventional specific treatment usually result in prompt and uneventful recovery. *P. falciparum* infection, drug toxicity and other infections may hamper the outcome. Special attention is needed when quinine is used for treatment because it may interfere with cyclosporine metabolism, decreasing its blood levels.

Screening of donors who have recently spent time (preceding 3 years) in malarious regions should be considered. Potential screening methods should include thick and thin smear stained with Giemsa. Rapid diagnostic tests detecting the HRP2 antigen can also be taken into consideration when an expert review of thick and thin smears is not available. Recipients traveling to endemic regions should be given appropriate chemoprophylaxis to prevent infection during travel. Chloroquine can potentiate levels of cyclosporine and appropriate dose adjustments should be made.

## Babesiosis

Babesiosis is a tick-borne, zoonotic protozoal illness that occurs after infection with *Babesia* spp., which invade and lyse red blood cells. Several species cause human disease and include *Babesia divergens* in Europe and *B. microti* in the USA. *B. divergens* appears to be more virulent than others. Transmission to humans occurs via ticks of the *Ixodes* genus or rarely through blood transfusion. There have been reports of concurrent transmission of infection with *Babesia* and *Borrelia burgdorferi* (Lyme disease agent) to the same host (usually an immunocompetent host), since the vector is the same tick.

Risk factors for severe babesiosis include anatomic or functional asplenia, immunocompromised state, rituximab use, thrombocytopenia, parasitemia >10%, and older age (>60 years). Only two cases have been reported to date after HSCT. 40,41 Clinical manifestations range from asymptomatic to life threatening disease. Early symptoms may be fever and malaise, which can progress to severe hemolytic anemia (potentially manifesting as a posttransplant hemolytic-uremic or hemophagocytic syndromes), adult respiratory distress syndrome, multi-organ failure and even death. Blood tests may show hemolytic anemia, thrombocytopenia and conjugated hyperbilirubinemia. Disease severity correlates with the degree of parasitemia, as in malaria.

Babesiosis can be diagnosed by direct microscopic visual review of peripheral blood smear or by NAAT of blood. Diagnostic confusion between *Plasmodium* spp. (malaria) and *Babesia* spp. can occur due to the similarity in morphology on microscopy when infecting red blood cells. Specific epidemiologic exposures and NAAT can aid in differentiating the diseases. For babesiosis bone marrow biopsy may reveal hemophagocytosis and marrow histiocytosis.

Babesiosis is a potentially life threatening infection in immunocompromised hosts and antimicrobial treatment should begin immediately. Exchange transfusion should be considered in cases of greater than 10% parasitemia, severe hemolysis, severe renal and/or hepatic and/or pulmonary involvement.^42^ Reduction in immunosuppressive regimen should be considered. Atovaquone (750 mg po q 12 h) plus azithromycin (500–1000 mg po on day 1, then 250–1000 mg/day po) for a total of 7–10 days can be used in those able to take oral medications (Table 2). Clindamycin (300 mg–600 mg iv qid × 7 days) plus quinine (650 mg po tid × 7–10 days) plus exchange transfusion (if >10% parasitemia) is recommended in severe cases. In a prospective, nonblinded, randomized trial of the two regimens in 58 normal hosts, atovaquone and azithromycin were as effective as clindamycin and quinine with fewer adverse reactions (15% vs. 72%). The most common adverse effects with atovaquone and azithromycin were diarrhea and rash (8% each), while clindamycin and quinine common adverse effects were tinnitus (39%), diarrhea (33%) and decreased hearing (28%). Azithromycin may increase the serum concentration of tacrolimus and patients should be monitored for toxicity. Sirolimus and tacrolimus metabolism may be slowed by the CYP3A4 inhibitor quinidine.

The optimal antimicrobial or combination therapy in transplant recipients is not clear; persistent relapsing illness has been well described in other immunocompromised hosts. In a series of 14 immunocompromised subjects, most with B-cell lymphoma, asplenic or treated with rituximab, anti-*Babesia* treatment was required for at least 6 weeks to achieve a cure. Resolution of persistent infection occurred in 11 patients when antibiotic treatment was continued ≥ 2 weeks after documenting negative blood smears. Three (21%) subjects died, highlighting the severity of disease in this population and the need for longer treatment and prolonged monitoring compared to normal hosts.^43^ Though rare, resistance to the atovaquone/azithromycin regimen can occur, more commonly in immunocompromised hosts.^44^ Blood smears should be used for monitoring response to therapy and for relapse after completion of treatment, NAAT can be considered when available.

When visiting endemic areas, transplant recipients should avoid tick exposure by using permethrin repellants on clothing, DEET or picaridin repellants on skin, and general protective clothing.^45^ Frequent tick checks and prompt removal is valuable, because early removal decreases the chance of transmission.

Prospective living donors should avoid high-risk exposures in endemic regions in the weeks prior to donation. Living donors with a prior diagnosis of *Babesia* infection should report this and document they are clear of infection prior to donation.

## Amebic Meningoencephalitis

**Figure 2:**
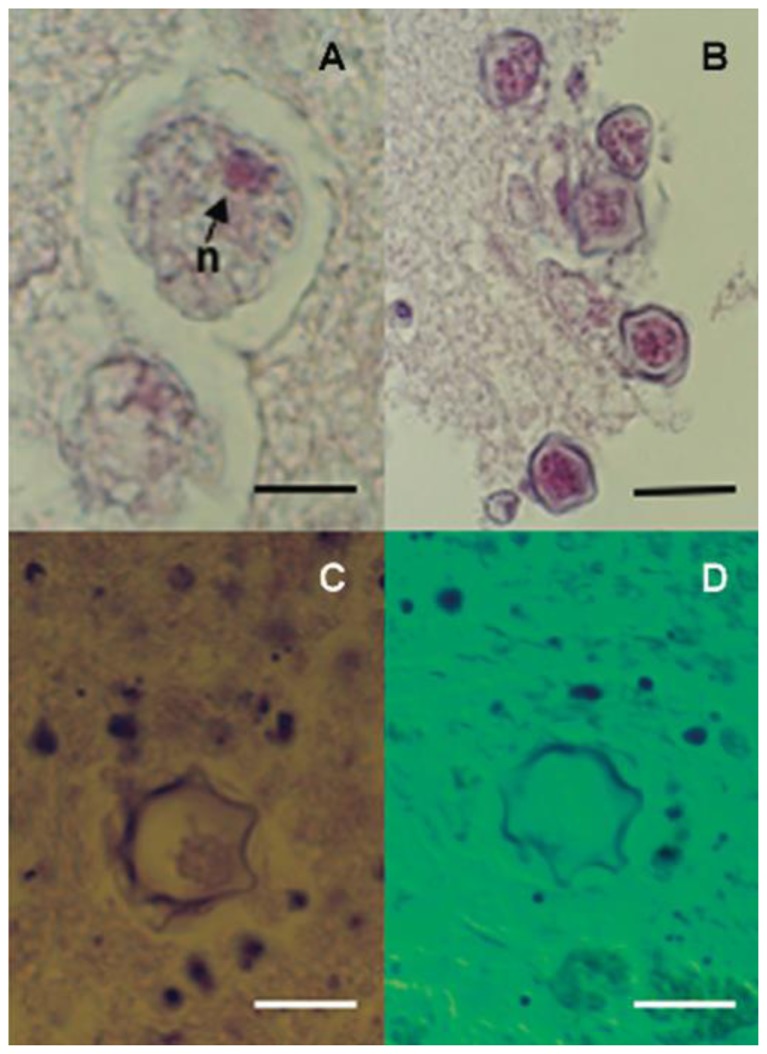
Histopathological observation of the brain tissue biopsy specimen showing *Acanthamoeba* trophozoite (A) and cysts (B, C, D) (hematoxylin-eosin staining); D: Nomarski differential interference contrast microscopy; n: nucleus; scale bars:A,C,D,10lm;B=20lm).

Acanthamoeba are protozoan parasites found in dust, soil, water, contact lens fluid, air conditioners, sewage, and may colonize the nose and throats of healthy individuals. Acanthamoeba can cause either focal disease (usually keratitis, granulomatous amebic encephalitis, brain abscess, pulmonary lesions, cutaneous lesions, or sinusitis) or disseminated acanthamebiasis that is often fatal in transplant recipients (Figure 2).^46^–^52^

Naegleria are ameba found in warm fresh water, heated contaminated tap-water, and soil. Infection can occur from exposition to contaminated water by swimming or by nasal sinus irrigation.

Diagnosis is made by identifying cysts or trophozoites in infected tissue. Cutaneous lesions may be the initial manifestation of infection and should be biopsied as early diagnosis is imperative to optimize the chance of survival. A direct examination of CSF should also be performed. Acanthamoeba can be cultured on agar plates coated with Gram-negative bacteria; it may take up to two weeks of culture before the amebas appear as track marks within the bacterial growth. Immunofluorescent tests may be used for species confirmation; DNA and RNA probes can also be used, but are not widely available. Serology is only useful for seroprevalence studies but not for diagnosis.

Optimal treatment regimens for Acanthamoeba infections remain unknown (Table 2). Drug sensitivities of free-living amebic infections differ between genera, species, and strains. Combinations of amphotericin B products with rifampin or imidazoles have been tried, as have combinations of sulfonamide antibiotics, azithromycin, miltefosine, caspofungin and flucytosine. Pentamidine has some *in vitro* activity. While some drugs are effective *in vitro* against *Naegleria*, nearly all infections are fatal. Prevention is the most important defense against this infection at the moment.

How best to prevent the rare infections due to Acanthamoeba is not clear, as the amoeba are fairly ubiquitous and seroprevalance rates are high. Cotrimoxazole has been used in treatment regimens; it is not known whether its common use in prophylaxis may be able to prevent infections. Naegleria prevention includes avoiding exposure. If nasal sinus irrigation is necessary, use boiled water, filtered (≤1 μm) water, or distilled/sterile water.

## Intestinal Parasitosis

Intestinal parasitic infections are prevalent in developing regions of the world. Accordingly, with increasing travel to and from endemic regions, intestinal parasites may have an increasingly significant role in transplantation. Moreover, relevant parasites including Strongyloides, Giardia, Cryptosporidium and Entamoeba have a worldwide distribution. A careful pretransplant social history can identify at-risk individuals who may benefit from focused screening for persistent parasitic infection. Intestinal parasitic infections are often asymptomatic before transplantation but flourish under immunosuppression, becoming clinically evident. Eosinophilia, gastroenteritis and other clinical manifestations of parasite infections prior to transplant should also trigger an appropriate workup.

### Intestinal protozoa

*Cryptosporidium, Cystoisospora belli*, Cyclospora, Microsporidia, *Blastocystis hominis* and Giardia can all cause significant, and sometimes protracted, gastroenteritis in transplant recipients.^53^ Transmission is more common in the developing world, with rates of infection as high as 20%, and can occur from contaminated food and water, person-to- person spread and zoonotic exposures. Cryptosporidium transmission in the developed world is facilitated by chlorine resistant oocytes and the 3–7 μm diameter of Cryptosporidium that can bypass many municipal water filtration and treatment systems. Moreover, infected individuals produce up to 100 million oocysts per day, while as few as 10–30 oocysts may cause infection in healthy persons.

Standard examination for ova and parasites in feces may be helpful but are time consuming. The concentration of stool and subsequent special stains may be more sensitive for certain pathogens; many laboratories use a modified trichrome stain or chromotrope 2R to diagnose microsporidial infections and modified acid fast stain for Cyclospora*.* EIA of stool may help rapidly diagnose Cryptosporidium and Giardia*.* Direct immunofluorescence tests for Giardia and Cryptosporidium are also available. NAAT may also be helpful when available. Electron microscopy of bowel biopsies may also be useful in diagnosing these infections and for Microsporidia species identification.

Intestinal protozoa infections are primarily acquired from contaminated food and water. Transplant recipients should avoid untreated well or lake water, and preferentially drink treated municipal water or bottled water. There are no data to support the use of bottled water over treated municipal water for transplant recipients. Person-to-person and zoonotic transmission can occur; transplant recipients should be aware of the potential risks.

The treatment of choice for intestinal parasitic infections in HSCT recipients is summarized in Table 2.

Strongyloidiasis is a parasitic disease caused by the nematode *Strongyloides stercoralis* that enter the body through exposed skin, such as bare feet. Strongyloidiasis is most common in tropical or subtropical climates and most people who are infected with *S. stercoralis* do not know they are infected and have no symptoms. Others may develop a severe form and, if untreated, become critically ill and potentially die. *S. stercoralis* infects approximately 100 million persons worldwide. The parasite is endemic in the tropics and subtropics, and has been reported from temperate areas such as Southern Europe. Strongyloides is found more frequently in the socioeconomically disadvantaged, in institutionalized populations, and in rural areas. It is often associated with agricultural activities. The most common way of becoming infected with Strongyloides is by contacting soil that is contaminated with Strongyloide*s* larvae. *S. stercoralis* is able to complete its life cycle both in the environment and in the human host. As a consequence, the parasite has an auto-infective cycle that produces long-term persistent infections. The rate of autoinfection is regulated by the immune response of the host and the severity of the disease correlates with worm burden. The major reservoir of the parasite is soil contaminated with human feces that harbor Strongyloides larvae. The filariform larvae penetrate the intact skin, enter the circulatory system, migrate to the lung, penetrate alveolar spaces, and move to the pharynx/trachea where swallowing allows access to the duodenal mucosa where they become adult parasites. Significant tissue phases of the life cycle accentuate blood eosinophilia. Adult females reproduce asexually (parthenogenesis) and sexually, laying eggs that become either rhabditiform larvae (which are eliminated with the stools completing the parasite life cycle), or filariform larvae that penetrate intestinal mucosa and perpetuate the infection. The molting of rhabditiform larvae into filariform larvae is accelerated under immunosuppression, allowing a massive number of larvae from the intestinal lumen or the perianal skin to autoreinfect the host. As a result, a high number of adult worms are found in the intestinal lumen. This can lead to lung involvement or the disseminated form of the disease.

Clinical presentation and outcomes depend on the interaction between the parasite and the host immune response, especially the Th-2 cell-mediated immunity. Some clinical conditions associated with defects in cell-mediated immunity can modify an asymptomatic intestinal carriage into a fulminant and frequently fatal disease.

Clinical syndromes include acute infection; chronic infection with parasite persistence and autoinfection manifested as localized gastrointestinal, cutaneous (*larva currens*) or pulmonary syndromes; hyperinfection syndrome (HIS) and disseminated disease (DD). HIS is characterized by accelerated larvae production, migration and elevated parasite burden with evident clinical manifestations, the larvae being restricted to pulmonary and gastrointestinal systems. DD includes the components of HIS with additional larva spread to other organs.^54^ Risk factors for HIS and DD have been linked to the immune status of the host and are mainly related to corticosteroids, anti-TNF agents, cytotoxic drugs or other immunosuppressive agents. Hypogammaglobulinemia and HTLV-I co-infection are also known risk factors for progression to HIS and DD.

Strongyloidiasis can be a devastating disease in transplant recipients; the mortality rate approaches 50% in HIS and 70% in DD. The clinical disease may present with pulmonary involvement, bacterial sepsis or bacterial meningitis with Gram-negative rods from intestinal flora carried on the surface of the parasite during tissue migration. Gastrointestinal presentations include acute and severe abdominal disease, bloody diarrhea, adynamic ileus, intestinal obstruction, and gastrointestinal hemorrhage, caused by larval damage inflicted as they penetrate through the gut wall. *S. stercoralis* HIS has been reported in recipients of HSCT, associated with high mortality rates.^55^–^57^ The risk is increased in allogeneic transplants in comparison with the autologous HSCT. In these patients, HS appears in the immediate post-transplantation period, when immunosuppression is most intense.^58^,^59^

**Figure 3:**
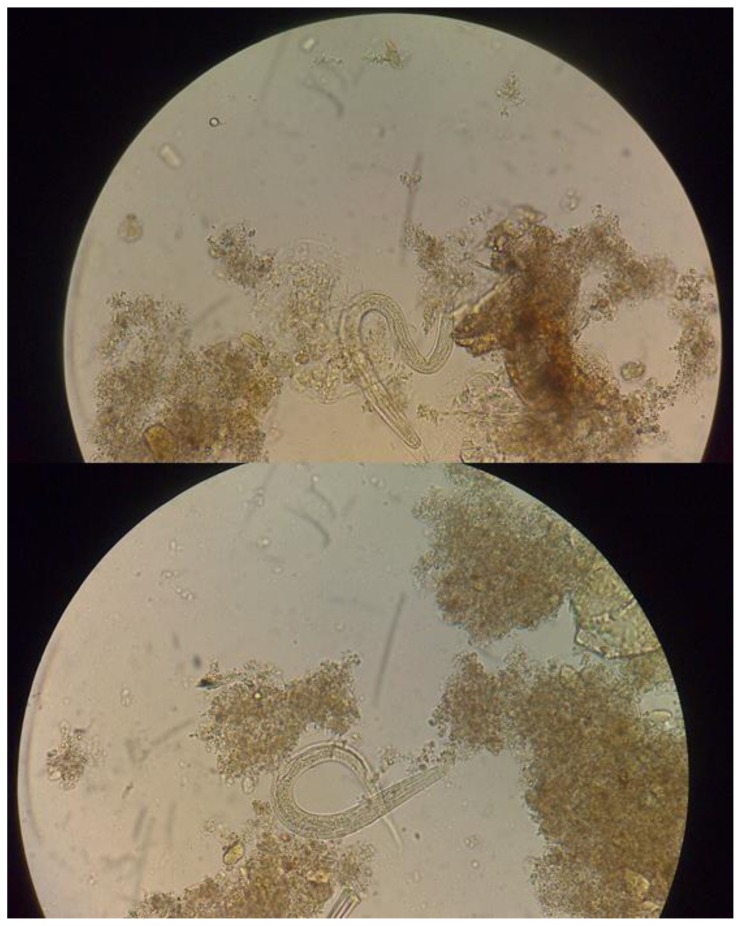
Rhabditiform larvae of *Strongyloides stercoralis* in stool (Wet mount 400X)

Eosinophilia can be found in patients with Strongyloides acute infection. However, patients with chronic infection, HIS, DD and immunocompromised patients may have normal eosinophil counts, even eosinopenia. The absence of eosinophilia does not rule out disease in recipient or donors.^60^ Definite diagnosis is achieved by identification of larvae by microscopic wet mount examination of liquid samples (mainly stool or duodenal aspirate) (Figure 3). Typically only HIS/DD have enough larvae to allow detection consistently.^61^ However, in the course of the DD larvae can be found in respiratory secretions, CSF, peritoneal fluid, urine, pleural effusion, blood and other tissue specimens. Larvae are often accidentally found when searching for other pathogens as causes of the severe disease. In uncomplicated cases, stool larvae density is low and elimination intermittent (direct observation sensitivity 0–14%). Duodenal fluid aspirate, while more sensitive than direct stool examination has only 76% sensitivity and involves an invasive procedure.

Serological testing is often more sensitive for diagnosis of infection, although cannot distinguish active and prior infection, and may not be available worldwide. Enzyme-linked immunosorbent assay (EIA) is highly sensitive (80–95%) and specific (90%) in normal hosts. In immunocompromised patients sensitivity is reduced to 68%, with retained specificity at 89%.^62^ False-positive results are mostly related to the presence of other helminthic infections; thus, local epidemiology is important when considering the positive predictive value. A gelatin particle indirect agglutination (GPIA) has 98% sensitivity and 100% specificity.

Ivermectin (200 μg/kg/day po × 2 days) is the treatment of choice for strongyloidiasis and is effective at eradicating adult parasites and larvae from the intestine in normal hosts with intestinal disease (Table 2). A repeated dose at two weeks is designed to treat the less susceptible forms by life cycle stage. Adverse effects are infrequent and usually mild. Albendazole (400 mg po bid × 7 days) has a primary cure rate of only 45–75% making it a second-line therapy. Thiabendazole, is the agent with the most clinical experience, although the least satisfactory of all available drugs, due to frequent relapses and toxicities.

The experience with ivermectin for the treatment of HIS or DD in transplant recipients is limited. Cases with heavy parasitic burden require daily doses until clearance; with additional doses for 7–14 days to reduce the risk of relapse. Anecdotal experiences lead some to advocate combination or sequential ivermectin and albendazole treatment. Severe strongyloidiasis with concomitant malabsorption is a serious challenge to oral treatment. Off-label rectal ivermectin can be effective in patients unable to tolerate or absorb oral therapy.^63^ A parenteral veterinary formulation of ivermectin has been used subcutaneously with some success.^64^

Transplant recipients should be educated to wear closed footwear in endemic environments to reduce the risk of primary infection. To mitigate the risk of disseminated strongyloidiasis in asymptomatic or paucisymptomatic patients, expanded screening with a detailed history, parasitological studies and serology facilitate necessary treatment of infection before transplantation is needed. In addition to asking about international travel, it is important to inquire about work, volunteer, or military service abroad which many patients do not consider “travel.” If not feasible, consider empiric treatment before initiation of immunosuppressive therapy for transplant candidates with unexplained eosinophilia, a history of parasitic infection and/or residence in or travel to, endemic areas even in the remote past.

Evaluation for latent strongyloidiasis should be strongly considered in transplant candidates with epidemiological risk factors or unexplained eosinophilia during pretransplant evaluation.

## Figures and Tables

**Table 1:** Main parasitic diseases in HSCT recipients.

Common name of disease	Organism	Involved sites	Diagnostic specimen/technique	Distibution/Prevalence	Source/Transmission (Reservoir/Vector)
Amebic meningo-encephalitis	*Naegleria fowleri* *Acanthamoeba* spp. *Balamuthia mandrillaris*	Brain, disseminated	Direct examination or Giemsa stain of CSF/brain to identify tissue cysts or trophozoites; Culture; NAAT	Rare but deadly	Nasal insufflations of contaminated warm fresh water, poorly chlorinated swimming pools, hot springs, soil
Babesiosis	*B. divergens, B. microti*	Red blood cells	Giemsa-stained thin blood smear, NAAT of whole blood	Different species have specific distribution: *B. divergens* (Europe), *B. microti* (USA)	Tick bites, e.g. *Ixodes scapularis* Blood transfusion
Blastocystosis	*Blastocystis* spp.	Intestine	Direct microscopy of stool, NAAT	Worldwide: one of the most common human parasites: USA, ~23% of the population; developing regions, 40–100% of the population	Eating food contaminated with feces from an infected human or animal
Chagas disease	*Trypanosoma cruzi*	Colon, esophagus, heart, nerves, muscle and blood	Serology Giemsa-stained thin blood smear NAAT of whole blood or tissue	Central America, South America: 16–18 million	Triatoma/Reduviidae - “Kissing bug” (insect vector feeds at night) Blood transfusion, infected mother to fetus, oral ingestion, or transplantation
Cryptosporidiosis	*Cryptosporidium* spp.	Intestine	Direct microscopy of stool, NAAT	Widespread	Ingestion of oocyst (sporulated), some species are zoonotic (e.g. bovine fecal contamination)
Cyclosporiasis	*Cyclospora cayetanensis*	Intestine	Direct microscopy of stool, NAAT	Widespread	Ingestion of oocyst through contaminated food
Isosporiasis	*Isospora belli*	Epithelial cells of small intestine	Direct microscopy of stool, NAAT	Worldwide - less common than *Toxoplasma* or *Cryptosporidium*	Fecal-oral route: ingestion of sporulated oocysts
Leishmaniasis	*Leishmania* spp.	Visceral (*L. donovani* complex): liver, spleen, bone marrow	Giemsa-stained bone marrow aspirate/biopsy, splenic aspirate, and NAAT	Visceral leishmaniasis: Worldwide	*Phlebotomus* or *Lutzomyia*- bite of several species of phlebotomine sandflies. Transfusion, transplantation or by sharing contaminated needles or syringes. Vertical transmission
Malaria	*Plasmodium falciparum* (80% of cases), *Plasmodium vivax, Plasmodium ovale, Plasmodium malariae* *Plasmodium knowlesi*	Red blood cells, liver, CNS	Giemsa-stained thick and thin blood smears, immunochromatographic assay and NAAT	Tropical - 300 million cases/year	Anopheles mosquito, bites at night Blood transfusions and organ transplantation
Strongyloidiasis	*Strongyloides stercoralis*	Intestine, lung, skin (*larva currens*)	Identification of larvae by direct microscopy of stool, sputum, CSF or duodenal aspirate; Serology	Worldwide, 100 millions persons	Skin penetration by contacting contaminated soil Auto-infestation
Toxoplasmosis	*Toxoplasma gondii*	Eye, brain, heart, liver	Serology Giemsa stain or NAAT in CSF or tissue	Worldwide: one of the most common human parasites; estimated to infect between 30–50% of the global population.	Ingestion of uncooked/undercooked pork/lamb/goat with *Toxoplasma* bradyzoites, ingestion of raw milk with *Toxoplasma* tachyzoites, ingestion of contaminated water food or soil with oocysts in cat feces

CFS, cerebrospinal fluid; CNS, central nervous system; NAAT, nucleic acid amplification tests

**Table 2:** Recommended treatment for parasitic infections in HSCT recipients.

Parasite	Treatment

Free-living amebae:	
*Acanthamoeba* spp.	Optimal treatment regimens remain unknown; combination therapy is essential and should include pentamidine, azoles, sulfonamides, and possibly flucytosine.
*Naegleria fowleri*	Therapy should include amphotericin B; consider intrathecal amphotericin. Combination systemic therapy is essential: consider addition of azoles, rifampin, or other antimicrobial agents.
*Balamuthia mandrilaris*	Combination therapy is essential and should likely include flucytosine, pentamidine, fluconazole, sulfadiazine, macrolides.

*Babesia* spp.	Atovaquone (750 mg po bid) plus azithromycin (500–1000 mg po on day 1, then 250–1000 mg/day po) × 7–10 days. Alternative therapy: Clindamycin 600 mg (pediatric: 20–40 mg/kg/day ) po tid or 1.2 g iv bid plus quinine 650 mg (pediatric: 30 mg/kg/day) po tid (or quinidine iv) to ≥2 weeks beyond clearance of parasitemia (≥6 weeks minimum total treatment).

*Blastocystis hominis*	Nitazoxanide (500 mg po bid × 3 days), metronidazole (1.5 g/day po × 10 days), iodoquinol (650 mg po tid × 20 days), or cotrimoxazole (1 tab bid × 7 days). Alternative therapy: Metronidazole 1.5 g daily × 10 days OR iodoquinol 650 g po tid × 20 days, OR cotrimoxazole (800/160) bid × 7 days

*Cryptosporidium* spp*.*	Nitazoxanide (500 mg po bid × 3 days), paromomycin, azithromycin, or combinations of these drugs.

*Cyclospora cayetanensis*	Cotrimoxazole (1 tablet po bid × 7–10 days), ciprofloxacin (500 mg po bid × 7 days, then 3 times a week × 2 weeks) or nitazoxanide are potential alternatives in the setting of significant sulfa allergy.

*Cystoisospora belli*	Cotrimoxazole (1 tablet po bid × 7–10 days), Ciprofloxacin (500 mg po bid × 7 days), pyrimethamine (50–75 mg/day po) combined with folinic acid (10–25 mg/day po) or nitazoxanide are potential alternatives in the setting of significant sulfa allergy.

*Giardia intestinalis*	Tinidazole (2 g po × 1 day), nitazoxanide (500 mg po bid × 3 days), metronidazole (250 mg po tid × 5–7 days), or paromomycin (10 mg/Kg po tid × 5–10 days); refractory disease: metronidazole plus quinacrine (100 mg po tid × 5 days).

*Leishmania donovani*	Liposomal amphotericin B (4 mg/kg daily on days 1–5, 10, 17, 24, 31 and 38, total dose of 40 mg/kg); consider secondary prophylaxis with intermittent dosing in patients at high-risk for relapse. Alternative therapy: Combination treatment with sodium stibogluconate plus miltefosine or paromomycin, but high toxicity.

Microsporidia	Albendazole (400 mg po bid × 2–4 weeks), fumagillin (20 mg po tid ×2 weeks).

*Plasmodium* spp.	*P. vivax*, *P. malariae*, *P. ovale* and uncomplicated *P. falciparum* infection in chloroquine-susceptible regions: chloroquine phosphate (1 g −600 mg base- po, them 0.5 g in 6 h, then 0.5 g daily × 2 days; total dose 2500 mg) Uncomplicated *P. falciparum* infection in a chloroquine-resistant region: artemisinin combination therapy (artemether-lumefantrine 4 tablets -80 mg/480 mg- as a single dose, then 4 tablets again after 8 h, then 4 tablets bid × 2 days). Severe cases of *P. falciparum* infection: intravenous artesunate followed by doxycycline, atovaquone-proguanil or mefloquine

*Schistosoma* spp.	Praziquantel 20 mg/kg/dose po bid × 1 day if *S. hematobium* or *S. mansoni*; praziquantel 20 mg/kg po tid × 1 day if *S. japonicum or S. mekongi*. Alternative therapy: Oxamniquine and artemether (anti-malarial).

*Strongyloides stercoralis*	Ivermectin (200 μg/kg/day po × 2 days); repeat in 2 weeks (3 mg tablets) (longer for hyperinfection); alternative therapy: Albendazole 400 mg po bid × 10–14 days (longer for hyperinfection); Hyperinfection: Treat until clearance – then 7–14 days longer. Off-label alternatives if oral therapy not an option: (a) Per rectum ivermectin (b) Subcutaneous ivermectin.

*Toxoplasma gondii*	Induction therapy (6 weeks) with pyrimethamine (200 mg po ×1 dose, then 75 mg/day po) (plus folinic acid) and sulfadiazine (1 g if <60 kg; 1.5 g if >=60 kg po, q6h) plus folinic acid (10–25 mg/day po). Alternative induction therapy: Pyrimethamine (same dosing as preferred therapy) plus clindamycin 600 mg iv/po qid OR cotrimoxazole (10 mg/kg TMP-50 mg/kg SMX) iv/po divided BID OR atovaquone 1500 mg po bid plus either pyrimethamine and leucovorin (same dosing as preferred therapy) or sulfadiazine (same dosing as preferred therapy) OR azithromycin 900–1200 mg po daily plus pyrimethamine and leucovorin (same dosing as preferred therapy).

*Trypanosoma cruzi*	Benznidazole (5–7 mg/kg/day po bid × 60 days); alternative therapy: Nifurtimox 8–10 mg/kg/day in 3 divided doses × 90 days.

## References

[1] Martín-Dávila P, Fortún J, López-Vélez R (2008). Transmission of tropical and geographically restricted infections during solid-organ transplantation. Clin Microbiol Rev.

[2] Schwartz BS, Mawhorter SD, AST Infectious Diseases Community of Practice (2013). Parasitic infections in solid organ transplantation. Am J Transplant.

[3] Saavedra S, Jarque I, Sanz GF (2002). Infectious complications in patients undergoing unrelated donor bone marrow transplantation: experience from a single institution. Clin Microbiol Infect.

[4] Parody R, Martino R, Rovira M (2006). Severe infections after unrelated donor allogeneic hematopoietic stem cell transplantation in adults: comparison of cord blood transplantation with peripheral blood and bone marrow transplantation. Biol Blood Marrow Transplant.

[5] Cahu X, Rialland F, Touzeau C (2009). Infectious complications after unrelated umbilical cord blood transplantation in adult patients with hematologic malignancies. Biol Blood Marrow Transplant.

[6] Kotton CN (2007). Zoonoses in solid-organ and hematopoietic stem cell transplant recipients. Clin Infect Dis.

[7] Martino R, Maertens J, Bretagne S (2000). Toxoplasmosis after hematopoietic stem cell transplantation. Clin Infect Dis.

[8] López-Duarte M, Insunza A, Conde E, Iriondo A, Mazorra F, Zubizarreta A (2003). Cerebral toxoplasmosis after autologous peripheral blood stem cell transplantation. Eur J Clin Microbiol Infect Dis.

[9] Power M, Vandenberghe E, Conneally E (2005). Retinal and cerebral toxoplasmosis following nonmyeloablative stem cell transplant for chronic lymphocytic leukaemia. Bone Marrow Transplant.

[10] Goebel WS, Conway JH, Faught P, Vakili ST, Haut PR (2007). Disseminated toxoplasmosis resulting in graft failure in a cord blood stem cell transplant recipient. Pediatr Blood Cancer.

[11] Duband S, Cornillon J, Tavernier E, Dumollard JM, Guyotat D, Péoc’h M (2008). Toxoplasmosis with hemophagocytic syndrome after bone marrow transplantation: diagnosis at autopsy. Transpl Infect Dis.

[12] Edvinsson B, Lundquist J, Ljungman P, Ringdén O, Evengård B (2008). A prospective study of diagnosis of Toxoplasma gondii infection after bone marrow transplantation. APMIS.

[13] Meers S, Lagrou K, Theunissen K (2010). Myeloablative conditioning predisposes patients for Toxoplasma gondii reactivation after allogeneic stem cell transplantation. Clin Infect Dis.

[14] Busemann C, Ribback S, Zimmermann K (2012). Toxoplasmosis after allogeneic stem cell transplantation--a single centre experience. Ann Hematol.

[15] Osthoff M, Chew E, Bajel A (2013). Disseminated toxoplasmosis after allogeneic stem cell transplantation in a seronegative recipient. Transpl Infect Dis.

[16] Montoya JG (2002). Laboratory diagnosis of Toxoplasma gondii infection and toxoplasmosis. J Infect Dis.

[17] Ionita C, Wasay M, Balos L, Bakshi R (2004). MR imaging in toxoplasmosis encephalitis after bone marrow transplantation: Paucity of enhancement despite fulminant disease. AJNR Am J Neuroradiol.

[18] Vidal CI, Pollack M, Uliasz A, del Toro G, Emanuel PO (2008). Cutaneous toxoplasmosis histologically mimicking graft-versus-host disease. Am J Dermatopathol.

[19] Beraud G, Pierre-Francois S, Foltzer A (2009). Cotrimoxazole for treatment of cerebral toxoplasmosis: An observational cohort study during 1994–2006. Am J Trop Med Hyg.

[20] Rodriques Coura J, de Castro SL (2002). A critical review on Chagas disease chemotherapy. Mem Inst Oswaldo Cruz.

[21] Villalba R, Fornés G, Alvarez MA (1992). Acute Chagas’ disease in a recipient of a bone marrow transplant in Spain: case report. Clin Infect Dis.

[22] Altclas J, Sinagra A, Jaimovich G (1999). Reactivation of chronic Chagas’ disease following allogeneic bone marrow transplantation and successful pre-emptive therapy with benznidazole. Transpl Infect Dis.

[23] Fores R, Sanjuan I, Portero F (2007). Chagas disease in a recipient of cord blood transplantation. Bone Marrow Transplant.

[24] Angheben A, Giaconi E, Menconi M (2012). Reactivation of Chagas disease after a bone marrow transplant in Italy: first case report. Blood Transfus.

[25] Bern C (2012). Chagas disease in the immunosuppressed host. Curr Opin Infect Dis.

[26] Le Loup G, Pialoux G, Lescure FX (2011). Update in treatment of Chagas disease. Curr Opin Infect Dis.

[27] Pinazo MJ, Miranda B, Rodriguez-Villar C (2011). Recommendations for management of Chagas disease in organ and hematopoietic tissue transplantation programs in nonendemic areas. Transplant Rev (Orlando).

[28] Molina I, Gómez i Prat J, Salvador F, Trevi-o B (2014). Randomized trial of posaconazole and benznidazole for chronic Chagas’ disease. N Engl J Med.

[29] Postorino MC, Bellantoni M, Catalano C (2011). Visceral leishmaniasis reactivation in transplant patients: A minireview with report of a new case. J Nephrol.

[30] Sirvent-von Bueltzingsloewen A, Rosenthal MP (2004). Visceral leishmaniasis: a new opportunistic infection in hematopoietic stem-cell transplanted patients. Bone Marrow Transplant.

[31] Agteresch HJ, van’t Veer MB, Cornelissen JJ, Sluiters JF (2007). Visceral leishmaniasis after allogeneic hematopoietic stem cell transplantation. Bone Marrow Transplant.

[32] Bautista G, Ramos A, Gil S (2012). Visceral leishmaniasis in hematopoietic stem cell transplantation. Transpl Int.

[33] Martinez-Losada C, Martin C, Cuenca T, Torres A (2013). Duodenal leishmaniasis after allogeneic hematopoietic SCT. Bone Marrow Transplant.

[34] Komitopoulou A, Tzenou T, Baltadakis J, Apostolidis J, Karakasis D, Harhalakis N (2014). Is leishmaniasis an “unusual” suspect of infection in allogeneic transplantation?. Transpl Infect Dis.

[35] Dantas Brito M, Campilho F, Branca R (2014). Visceral leishmaniasis: A differential diagnosis to remember after bone marrow transplantation. Case Rep Hematol.

[36] Miller CE, Bain BJ (2015). The utility of blood and bone marrow films and trephine biopsy sections in the diagnosis of parasitic infections. Mediterr J Hematol Infect Dis.

[37] Reithinger R, Dujardin JC (2007). Molecular diagnosis of leishmaniasis: Current status and future applications. J Clin Microbiol.

[38] Vigna E, De Vivo A, Gentile M (2010). Liposomal amphotericin B in the treatment of visceral leishmaniasis in immunocompromised patients. Transpl Infect Dis.

[39] Jarvis JN, Lockwood DN (2013). Clinical aspects of visceral leishmaniasis in HIV infection. Curr Opin Infect Dis.

[40] Cirino CM, Leitman SF, Palmore TN (2008). Transfusion-associated babesiosis with an atypical time course after nonmyeloablative transplantation for sickle cell disease. Ann Intern Med.

[41] Lubin AS, Snydman DR, Miller KB (2011). Persistent babesiosis in a stem cell transplant recipient. Leuk Res.

[42] Kappagoda S, Singh U, Blackburn BG (2011). Antiparasitic therapy. Mayo Clin Proc.

[43] Krause PJ, Gewurz BE, Hill D (2008). Persistent and relapsing babesiosis in immunocompromised patients. Clin Infect Dis.

[44] Wormser GP, Prasad A, Neuhaus E (2010). Emergence of resistance to azithromycin-atovaquone in immunocompromised patients with Babesia microti infection. Clin Infect Dis.

[45] Carroll JF, Benante JP, Kramer M, Lohmeyer KH, Lawrence K (2010). Formulations of deet, picaridin, and IR3535 applied to skin repel nymphs of the lone star tick (Acari: Ixodidae) for 12 hours. J Med Entomol.

[46] Anderlini P, Przepiorka D, Luna M (1994). Acanthamoeba meningoencephalitis after bone marrow transplantation. Bone Marrow Transplant.

[47] Feingold JM, Abraham J, Bilgrami S (1998). Acanthamoeba meningoencephalitis following autologous peripheral stem cell transplantation. Bone Marrow Transplant.

[48] Castellano-Sanchez A, Popp AC, Nolte FS (2003). Acanthamoeba castellani encephalitis following partially mismatched related donor peripheral stem cell transplantation. Transpl Infect Dis.

[49] Pemán J, Jarque I, Frasquet J (2008). Unexpected postmortem diagnosis of Acanthamoeba meningoencephalitis following allogeneic peripheral blood stem cell transplantation. Am J Transplant.

[50] Kaul DR, Lowe L, Visvesvara GS, Farmen S, Khaled YA, Yanik GA (2008). Acanthamoeba infection in a patient with chronic graft-versus-host disease occurring during treatment with voriconazole. Transpl Infect Dis.

[51] Akpek G, Uslu A, Huebner T (2011). Granulomatous amebic encephalitis: an under-recognized cause of infectious mortality after hematopoietic stem cell transplantation. Transpl Infect Dis.

[52] Doan N, Rozansky G, Nguyen HS (2015). Granulomatous amebic encephalitis following hematopoietic stem cell transplantation. Surg Neurol Int.

[53] Marcos LA, Gotuzzo E (2013). Intestinal protozoan infections in the immunocompromised host. Curr Opin Infect Dis.

[54] Keiser PB, Nutman TB (2004). Strongyloides stercoralis in the immunocompromised population. Clin Microb Rev.

[55] Orlent H, Crawley C, Cwynarski K, Dina R, Apperley J (2003). Strongyloidiasis pre and post autologous peripheral blood stem cell transplantation. Bone Marrow Transplant.

[56] Schaffel R, Portugal R, Maiolino A, Nucci M (2004). Strongyloidiasis pre and post autologous peripheral blood stem cell transplantation. Bone Marrow Transplant.

[57] Qazilbash MH, Ueno NT, Hosing C (2006). Strongyloidiasis after unrelated nonmyeloablative allogeneic stem cell transplantation. Bone Marrow Transplant.

[58] Wirk B, Wingard JR (2009). Strongyloides stercoralis hyperinfection in hematopoietic stem cell transplantation. Transpl Infect Dis.

[59] Ramanathan R, Nutman T (2008). Strongyloides stercoralis infection in the immunocompromised host. Curr Infect Dis Rep.

[60] Vadlamudi RS, Chi DS, Krishnaswamy G (2006). Intestinal strongyloidiasis and hyperinfection syndrome. Clin Mol Allergy.

[61] Siddiqui AA, Berk SL (2001). Diagnosis of Strongyloides stercoralis infection. Clin Infect Dis.

[62] Schaffel R, Nucci M, Carvalho E (2001). The value of an immunoenzymatic test (enzyme-linked immunosorbent assay) for the diagnosis of strongyloidiasis in patients immunosuppressed by hematologic malignancies. Am J Trop Med Hyg.

[63] Tarr PE, Miele PS, Peregoy KS, Smith MA, Neva FA, Lucey DR (2003). Case report: Rectal administration of ivermectin to a patient with Strongyloides hyperinfection syndrome. Am J Trop Med Hyg.

[64] Marty FM, Lowry CM, Rodriguez M (2005). Treatment of human disseminated strongyloidiasis with a parenteral veterinary formulation of ivermectin. Clin Infect Dis.

